# Editorial: ‘Engineering the Tumor Immune Microenvironment’ Special Issue

**DOI:** 10.3390/cancers15164014

**Published:** 2023-08-08

**Authors:** Raffae N. Ahmad, Scott S. Verbridge

**Affiliations:** Department of Biomedical Engineering and Mechanics, Virginia Tech, Blacksburg, VA 24061, USA; raffae@vt.edu

Cancer immunotherapies, while promising and occasionally even curative, encounter numerous hurdles within the tumor microenvironment that hinder their efficacy. The presence of an immunosuppressive tumor immune microenvironment (TIME) significantly limits the effectiveness of targeted immunotherapies. To maximize the potential of cancer immunotherapies across different malignancies, it is crucial to expand our understanding of the TIME and develop innovative techniques to overcome the obstacles contained therein. Interdisciplinary approaches are particularly appealing as known barriers include both biological as well as physical hallmarks of tumors. This Special Issue of *Cancers* is a collection of 13 impactful papers, comprising 7 original articles and 6 reviews. These contributions delve into emerging engineering approaches for immunotherapies, the characterization and modification of the TIME, and comprehensive reviews addressing diverse aspects of these topics. We will highlight several of these works below, and encourage the reader to refer to the Special Issue for additional details. 

Some of the most impressive immunotherapy outcomes have been observed in the treatment of leukemias; however, major challenges and unpredictable variations in response remain. It is well established that dysregulation of epigenetic activation and silencing is a hallmark of acute myeloid leukemia (AML). Hypermethylating agents are the standard of care for the treatment of AML, although resistance can develop, resulting in disease relapse. Using immune-deficient and immune-proficient AML mouse models, Ebelt and Manuel [1] highlight the effect of 5-Azacytidine treatment on the expression of checkpoint proteins in both cancer and immune cell populations, as well as the increased survival of mice following treatment. Solid tumors, in contrast, remain largely intractable to immunotherapy. As one potential path forward, Zamloot et al. [2] engineered an attenuated *Salmonella typhimurium* (ST) secreting a bacterial hyaluronidase (YS-HAse) in order to overcome the excessive stromal matrix components typically found within breast tumors. ST has an innate ability to preferentially colonize solid tumors, while carrying a highly controllable plasmid, allowing for targeted delivery of bacterial hyaluronidase with minimal systemic toxicity. In an orthotopic breast tumor mouse model, this team’s work demonstrates that administration of YS-HAse resulted in the colonization of the tumor and enabled the control of tumor growth through the degradation of intratumoral hyaluronic acid. 

Myeloid-derived suppressor cells (MDSCs) play a pivotal role in the reorganization of the extracellular matrix (ECM) and the establishment of an immunosuppressive TIME, thereby promoting a pro-metastatic state. In their article, Roberts et al. [3] investigated the response of MDSCs to interstitial fluid flow (IFF) within the TIME of breast cancer using an innovative 3D platform. The study revealed that while sorted GR1+ splenocytes exhibited reduced migration compared with the unsorted population, the presence of 4T1 mammary carcinoma cells and lymphatic endothelial cells led to increased migration of MDSCs in the presence of IFF. Furthermore, through inhibition of VEGFR3, the team demonstrated a significant decrease in IFF-driven invasion of both MDSCs and 4T1 cells in the co-culture model. This work, therefore, provides further insights into the complex interplay of biological and physical cues, which may promote an immunosuppressive TIME while also revealing potential therapeutic vulnerabilities. 

In another contribution, Dunn et al. [4] report a novel method for the time-course monitoring of cells in the peritoneal cavity of mice through minimally invasive in vivo intraperitoneal lavage (IVIPL). Using this method, the authors studied intraperitoneal cell populations over the course of CAR-T cell therapy in an ovarian xenograft murine model. They found ovarian cancer cells (OVCAR8-FG) to upregulate PD-L1, while human CAR+ therapeutic cells upregulated inhibitory factors PD-1 and TIM-3. The authors also investigated the intraperitoneal TIME in a syngeneic ID8 ovarian cancer murine model. Using this model, they analyzed cell populations during tumorigenesis, noting significant increases in tumor-associated macrophages and trends toward decreased NK1.1+ and CD8+:CD4+ T-cell ratios. This type of dynamic and high-resolution monitoring of immunotherapy response could ultimately provide valuable insights to improve efficacy and response consistency for patients. 

The remaining original studies included in this issue focus on other means of targeting therapy resistance to enhance treatment efficacy. For instance, Park et al. [5] demonstrate that tumor-derived extracellular vesicles may inhibit the activity of antitumor immune cells. They further explore the means through which such inhibition may be reversed in a targeted manner and combined with more traditional checkpoint inhibitors. Li et al. [6] provide an alternative route to reversing local cell-driven immunosuppression, specifically by targeting tumor-associated macrophages. Sailer et al. [7] explore related themes, focusing on the design of even more effective antitumor T cells using a novel multi-receptor approach. 

In addition to these new experimental studies, several insightful review papers are also presented. In their comprehensive review, Vitorino et al. [8] examine the complex microbiota present within the TIME of breast cancer and explore the impact of dysbiosis, an alteration in the microbiota composition, on disease progression. This review emphasizes the existence of tumor-type-specific microbiota and underscores the richness and diversity of the microbiota within the breast TIME. Furthermore, the authors delve into the close association between the immune system and microbiota, shedding light on their interactions with therapeutic agents and their implications for treatment efficacy and toxicity. This comprehensive analysis provides valuable insights into the intricate interplay between microbiota and breast cancer, opening avenues for further research and therapeutic exploration.

In their systematic review, Pal and Sheth [9] highlight the impact of biophysical factors within the TIME, such as interstitial fluid pressure, dense ECM, and collapsed intratumoral vessels, which can significantly reduce the efficacy of both chemotherapies and immunotherapies. The authors explore emerging technologies such as ultrasound, photodynamic therapy, electrotherapy, radiation, nanotechnology, and thermal-based modalities as potential approaches to modulate the TIME. Emphasizing the significance of combinatorial therapies, novel drug delivery methods, and diverse ablation technologies, the authors underscore the necessity to overcome these biophysical challenges.

Additional review topics include an investigation of the barriers faced by adoptive T-cell therapies in solid tumors, which is presented by Fuchsl and Krackhardt [10], as well as considerations of the role of perhaps lesser-known components of the TIME, such as tertiary lymphoid structures discussed by Vaghjiani and Skitzki [11], the role of the stromal microenvironment in multiple myeloma explored by Solimando et al. [12], and the role of microglia and immunosuppression in glioblastoma (GBM), discussed by Mormino and Garofalo [13]. We are hopeful that these reviews will help to point the way forward, contributing to the development of new therapies or approaches for these highly refractory tumor types. 

The TIME presents a critical barrier for existing therapies, but it also provides a potential target opportunity for more effective next-generation immunotherapies. To tackle both the biological as well as physical hurdles of the TIME head-on, we believe this field is poised to make great strides by embracing a fully interdisciplinary approach. We hope the articles amassed in this Special Issue may suggest that, with a better understanding and control of the TIME, researchers and clinicians can ultimately develop a new paradigm for effective antitumor management that can be of benefit to the majority of patients.
